# Retention of medical doctors at the district level: a qualitative study of experiences from Tanzania

**DOI:** 10.1186/s12913-018-3059-0

**Published:** 2018-04-10

**Authors:** Nathanael Sirili, Gasto Frumence, Angwara Kiwara, Mughwira Mwangu, Amani Anaeli, Tumaini Nyamhanga, Isabel Goicolea, Anna-Karin Hurtig

**Affiliations:** 1 Unit of Epidemiology and Global Health, Department of Public Health and Clinical Medicine, Umeå University, Sweden, SE90185 Umeå, Sweden; 2 Department of Development Studies, School of Public Health and Social Sciences, Muhimbili University of Health and Allied Sciences, P.O.BOX 65454 Dar es Salaam, Tanzania

**Keywords:** Physicians, Retention, Medical doctors, Rural, Africa, Health workforce, Human resources, Health systems, Tanzania, Decentralization

## Abstract

**Background:**

Retention of Human Resources for Health (HRH), particularly doctors at district level is a big challenge facing the decentralized health systems in poorly resourced countries. Tanzania, with 75% of its population in rural areas, has only 26% of doctors serving in rural areas. We aimed to analyze the experiences regarding the retention of doctors at district level in Tanzania from doctors’ and district health managers’ perspectives.

**Methods:**

A qualitative study was carried out in three districts from June to September 2013. We reviewed selected HRH documents and then conducted 15 key informant interviews with members of the District Health Management teams and medical doctors working at the district hospitals. In addition, we conducted three focus group discussions with Council Health Management Team members in the three districts. Incentive package plans, HRH establishment, and health sector development plans from the three districts were reviewed. Data analysis was performed using qualitative content analysis.

**Results:**

None of the districts in this study has the number of doctors recommended. Retention of doctors in the districts faced the following challenges: unfavourable working conditions including poor working environment, lack of assurance of career progression, and a non-uniform financial incentive system across districts; unsupportive environment in the community, characterized by: difficulty in securing houses for rent, lack of opportunities to earn extra income, lack of appreciation from the community and poor social services.

Health managers across districts endeavour to retain their doctors through different retention strategies, including: career development plans, minimum financial incentive packages and avenues for private practices in the district hospitals. However, managers face constrained financial resources, with many competing priorities at district level.

**Conclusions:**

Retention of doctors at district level faces numerous challenges. Assurance of career growth, provision of uniform minimum financial incentives and ensuring availability of good social services and economic opportunities within the community are among important retention strategies.

**Electronic supplementary material:**

The online version of this article (10.1186/s12913-018-3059-0) contains supplementary material, which is available to authorized users.

## Background

Human Resources for Health (HRH) has been defined as all people engaged in actions whose primary intent is to enhance health [1]. Globally, the retention of HRH has attracted attention because of the observed global geographical HRH imbalance [2]. Sub-Saharan Africa, home to 11% of the global population and 24.3% of the global burden of diseases, accounts for only 3% of the total global HRH [2]. Conversely, the USA and Canada, home to 15% of the global population and 13% of the global burden of diseases, host 37% of the global HRH [2]. Despite the observed regional imbalance, retention of the HRH – and particularly of physicians in rural areas – remains a big challenge facing policy- and decision-makers of today within countries worldwide [3–5]. Approximately one half of the global population lives in rural areas and is served by less than a quarter of the total physician HRH [6, 7].

Sub-Saharan Africa (SSA) suffers from the challenge of retaining its physicians (medical doctors) from both the international brain drain and the rural–urban migration within countries [2, 8]. The international brain drain of physicians from SSA is fuelled by both push factors in SSA countries and pull factors in the receiving countries, which are mostly high-income countries [2, 9]. The push factors include low financial compensation and unsatisfactory working conditions coupled with poor retention mechanisms [10, 11]. The pull factors in the receiving countries include good salaries, good working conditions and good future prospects in academia and research [2, 8]. Within the countries, poor working conditions and poor social services in rural areas have contributed to the rural–urban migration [11, 12].

### Retention of medical doctors (MDs) in Tanzania

Like other parts of SSA, Tanzania suffers from both a shortage and maldistribution of its physicians [13]. The doctor-to-population ratio in 2012 was estimated at 1:20,000 [14]. This ratio is far below the ratio recommended by World Health Organization (WHO) of 1:10,000 [15] and was not uniformly distributed within the country [16]. Only 31% of the MDs were serving the rural population, which accounted for over 75% of the total population [17]. It is further estimated that the percentage of MDs working in rural Tanzania decreased to 26% in 2015 [18]. This happened despite the increase in the number of MDs graduating annually from the training institutions, following the 1990s health sector reforms in Tanzania, from less than 50 MDs in 1990s to more than 350 MDs in 2010 [13, 19].

The 1990s health sector reforms opened an avenue for the opening up of private institutions training MDs as well as other health professions [20]. Post the reforms, the government has continued to sponsor the training of MDs even in private institutions through public-private partnership [20, 21]. Under this arrangement the government covers all costs payable direct to the institutions and provides grants to students to cater for meals, accommodation, fieldwork and stationery [21]. Following the government sponsorship of medical students, after graduation the MDs are bonded to practise for a minimum of five years within the country [21].

The observed maldistribution of MDs in Tanzania is contrary to what was expected after the introduction of the decentralized health sector administration system, which was part of the 1990s health sector reforms [22]. The decentralized administration system, which aimed at reducing the geographical imbalance of MDs among other HRH, gave mandates for: health-care programme planning, implementation, hiring and firing of MDs among other HRH within the district authorities [23]. Contrary to what was expected, the decentralized system failed to ensure the retention of MDs at the district level [24]. Therefore, in 2006 another system of HRH management, which combines centralization and decentralization and is known as a partial-centralized system, was introduced [24]. In the partial-centralized system, the local government identified vacancies and the central government filled them [24]. In 2008 the government introduced the first HRH strategic plan, the 2008–2013 HRH Strategic Plan [16]. Among other things, this strategic plan outlined strategies for establishing equitable distribution of doctors in the country by ensuring deployment to and retention in areas with critical shortage.

The aim of this work was to study experiences regarding the retention of MDs at the district level under the decentralized or partial-centralized health-care sector from the perspective of MDs and health managers. We focused our study on the retention of MDs for two main reasons: first, the training and deployment of MDs post-1990s health sector reforms has received considerable attention and consumes a lot of government resources [13, 20, 21]; however, regardless of this attention to the training and deployment system, the number of MDs at the district level has remained stagnant and in some instances has decreased [16, 25]. Second, most of the studies on the retention of HRH in rural Tanzania have either focused on only absolute numbers of all cadres, only health workers’ perspectives, only health managers’ perspectives, or the views of health workers and managers in a single district without comparing contexts, and we did not find any study focused specifically on doctors’ retention in Tanzania [25–28].

## Methods

### Study design

We adopted a qualitative study design in which we used key informant interviews (KIIs), focus group discussions (FGDs), and a review of documents for data collection. Qualitative study design was appropriate as retention of doctors in the decentralized health sector involves social processes and is dependent on the context [29].

### Study setting

Tanzania’s health-care sector operates in a pyramid with three levels of health-care services provision: primary, secondary and tertiary levels [22]. The primary level is composed of the district hospitals and all facilities below them. The secondary level is comprised of regional hospitals and regional referral hospitals and the tertiary level contains the national hospital, zonal referral hospitals and consultant hospitals. Each higher level acts as a referral to its immediate lower level. Tanzania mainland is subdivided into 7 geopolitical zones, 25 administrative regions and 159 districts [17].

This study was conducted between June and September 2013 in three districts of Tanzania mainland, two located in the eastern zone and one in the western zone. The two districts in the eastern zone were labelled as A and C while that in the western zone was labelled as B. The three districts were purposefully selected to capture a wide range of experiences regarding the retention of doctors due to the variation of the doctor to population ratio in the country [30].

District A is one of the municipalities in a cosmopolitan region in the eastern zone – it has the largest doctors-to-population ratio in that zone. District C is one of the hardest districts to reach due to limited means of transport and is in one of the regions forming the eastern zone – it has the smallest doctors-to-population ratio in the eastern zone. District B is an urban district with no district hospital in one of the regions in the western zone – its doctors-to-population ratio is on average similar to other districts in the western zone.

### Data collection

We started by a review of documents to familiarize ourselves with the context of the shortage of doctors and the overall HRH crisis in each district and the strategies available to address the shortage. The documents reviewed included: the district health system staffing norm; the incentive package plan; the career development programme; HRH Strategic Plan 2008–2013; and the country HRH profile reports.

We then conducted 15 key informants interviews (KIIs) with nine medical doctors (MDs) and six health managers (three district medical officers (DMOs) and three district health secretaries (DHSs)) and three focused group discussions (FGDs) with 22 members of the Council Health Management Teams (CHMTs) from the three districts (Table 1). The CHMT is composed of eight permanent members; however, it also has several co-opted members who may be invited to attend the CHMT meetings, depending on the issues under discussion. The eight CHMT members are; District Medical Officer, District Health Secretary, District Dental Officer, District Nursing Officer, District Health Officer, District Pharmacist, District Laboratory Technician and District Social Welfare Officer. We interviewed all available MDs in district B and C owing to the small number of MDs in these districts, while in District A, owing to its large number of MDs, we purposefully selected MDs based on their duration of working in the district to capture a wide range of experiences. Although district A had 59 MDs we stopped data collection after the seventh interview after attaining information saturation, as our aim was not generalizability of the findings but to analyse the qualitative experiences from the three settings. To carry out the KIIs, we used an interview guide that we developed based on the literature on the HRH crisis and the challenges faced in retaining HRH in Tanzania (See Additional file 1).

**Table 1:** Summary of key informants and FGD participants from the three districts

District	No. of KIIs	No. of FGD participants
A	7 (5 MDs and 2 Health Managers)	7
B	4 (2 MDs and 2 Health Managers)	9
C	4 (2 MDs and 2 Health Managers)	6
Total	15	22

Each interview was conducted by the first author in the office of the informant and lasted between one and two hours; each interview was audio-recorded using a digital audio recorder, and during the interview a research assistant who accompanied the first author took notes.

We prepared an FGD guide based on the existing literature on the HRH crisis and challenges of retaining HRH in Tanzania to carry out FGDs in the three districts. The appointment to meet the CHMT members was set by the DHS of each particular district. We conducted one FGD in each of the three districts with the aim of capturing rich and shared experiences on the retention of MDs in the districts, as this is a complex phenomenon cutting across many departments. The FGDs were conducted in one of the rooms at the district health offices. The first author moderated the FGDs, each FGD was audio-recorded using a digital audio recorder and during the discussion, a research assistant who accompanied the first author took notes. Each FGD lasted between one hour and one hour and thirty minutes.

### Data analysis

Audio-recorded interviews and FGDs were first transcribed verbatim and then translated from Swahili to English. We analysed the interviews and FGD transcripts using qualitative content analysis following Graneheim and Lundman [31]. Qualitative content analysis offers development of categories from the text data inductively; the inductive derivation of categories is important in capturing the experiences of the participants [32].

The full transcripts and field notes were first read and reread by all authors in order for them to become familiarized with the data and the context. Condensed-meaning units were then formed through data reduction. These were related to experiences on the retention of MDs in the districts from the perspectives of MDs and health managers. The condensed-meaning units were read and reread in order to extract the codes. The first and second authors extracted primary codes; these were shared, discussed, revisited and final codes agreed by all authors. Similar codes were grouped together and through constant comparison were abstracted into subcategories. Subcategories were further analysed to distinguish their similarities and differences. Similar subcategories were sorted to form categories that reflected the manifest content of the text.

### Ethical considerations

Muhimbili University of Health and Allied Sciences granted ethical clearance for this study (reference numbers are; 2012–09-03/IRB/VOL.III/03 and 2015–09-04/AEC/VOL. X/01). Permission to conduct the study was obtained from MoHSW, the District Executive Director, the Municipal Director, the Town Council Director, and District Medical Officers. Written informed consent was obtained from each informant before commencing the interview or discussion.

## Results

From the analysis of documents, none of the three districts had the minimum number of MDs recommended for their staffing level. District A had 59 out of 80 required MDs, equivalent to a 26.3% shortage. Districts B and C had 3 out of 8 required MDs in each district, the shortage of which accounted for 62.5%.

**Fig. 1:**
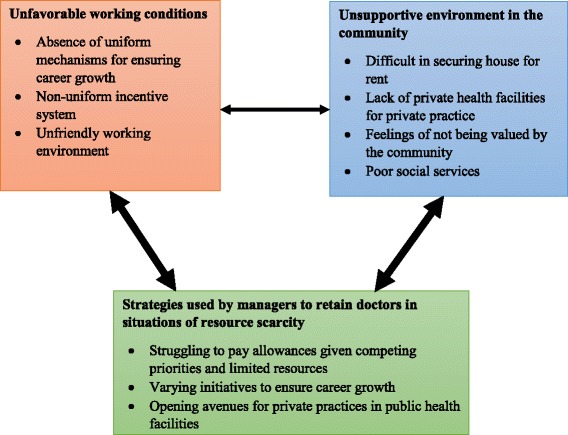
Summary of findings illustrating how the three categories are related to one another and the subcategories within each category

Three categories – unfavourable working conditions, unsupportive environment in the community, and retention strategies by the managers in situations of resource scarcity – emerged from the interviews and focused group discussions in this study (Fig. 1).

### Unfavourable working conditions

Unfavourable working conditions characterized by the absence of uniform mechanisms to ensure career growth, a non-uniform financial incentive system, and unfriendly work environments were found to make the retention of MDs difficult across the districts.

From the three districts, we found that there was no uniform mechanism in ensuring career growth. We found that while managers from all three districts recognized the importance of career growth in the retention of MDs, district C had no written career development plan. Districts A and B had career development plans that differed widely in terms of their implementation. This variation in career development plans across districts was explained to stimulate the MDs to find ways of relocating from one district to another where their career growth would be assured.

Informants from this study revealed that each district had its own financial incentive system for the MDs. District A had four different forms of allowances; district B had two, while district C had only one form of allowance [Table 2]. From the interviews we found that regardless of the existence of various forms of financial incentives, only district A was capable of effecting its financial incentive package on time. In districts B and C, payment of the financial incentives was associated with prolonged delays of sometimes more than a year. MDs interviewed from Districts B and C wished to move to other districts where there were good financial incentives that were paid on time.

‘*When I reported here I was told I would be provided with a housing allowance of Tshs 80,000/ month… I am staying in a house where the rent is Tshs 200,000/month…but since I came here more than a year ago I have not received that 80,000 even for a single month…. If I get another position where I will be valued, I will leave this place…*’ [medical doctor-District B]

**Table 2:** Minimum financial incentive package in each district

District	Forms and amount of allowances
A	On call (20,000 TZS per call), Housing (150,000 TZS per month), Uniform allowance (varies), and Responsibility allowance for head of sections (varies)
B	On call (20,000 TZS per call), Housing (80,000 TZS per month)
C	On call (20,000 TZS per call)

Unfriendly work environments, attributable to the lack of a district hospital in District B, the underequipped district hospital in District C and the lack of reliable means of transport for referral in District C were mentioned as affecting the retention of MDs in Districts B and C.

Due to the lack of a district hospital in District B, its MDs were attached to the regional hospital while receiving their payments from the district authorities. The presence of MDs from regional and district authorities performing similar roles in the same hospital but receiving different incentives was stated as among the factors that stimulate some MDs from District B to plan to relocate to other places. In District C, MDs stated that the district hospital was underequipped to such an extent that it was discouraging them to stay on, and hence their intention to leave.

*‘As a trained doctor, I really need to practise what I was trained for… Here I am utilizing a very small portion of my knowledge... Imagine I rely on very basic laboratory investigations and X-ray to come to the conclusion of a complicated diagnosis… The chance of false diagnosis is great… Even when you have the right diagnosis, again, the lack of medicine challenges you… I wish to be in a place where I can utilize at least 50% of my knowledge…’ [medical doctor-District C]*

The MDs in District C stated that they were sometimes attending patients that were in critical condition and were initially referred to higher-level facilities but failed to go due to the absence of reliable means of transport. This situation was bringing dissatisfaction to the MDs, discouraging them from staying, and hence intending to leave.

*‘You give a referral to a patient but they cannot afford the transport costs… Normally it is required that when you refer a patient you must provide a vehicle and an escort nurse… How can we do that in this case? ...The cost of a flight is very high…it is really discouraging… Most of the time we end up witnessing and certifying the death of patients who we referred before but who failed to go...’ [medical doctor-District C]*

### Unsupportive environment in the community

Poor physical and social support in the community attributed to MDs having difficulty in securing houses for rent, the lack of health facilities for private practice to earn extra income, MDs feeling that they were not valued by the community, and poor social services such as education and recreational facilities was another challenge facing the retention of MDs in all three districts.

We found that none of the three districts had its own houses for the MDs. This made the accommodation of MDs very difficult, especially when they first arrived. Some had to stay with their relatives or friends if they had some in those areas, while the majority had to face the high lodging costs.

Although the housing problem existed across the three districts, its magnitude varied widely. District C was most affected to the extent that even securing a house for renting from the community was difficult due to a lack of trust by the community. This situation made the MDs in District C feel as if they were not needed there and, hence they searched for opportunities elsewhere in order to leave.

‘*Life is very difficult here... If you can imagine, the indigenous treat us as foreigners who are here for our own interests… It is very difficult to rent a house here, until you have stayed here for a long period… Just as others that were here before, I am also on my way of leaving…*’ [health manager-District C]

The presence of private facilities that offered opportunities for part-time (after work in the public sector) practice for earning extra income on top of their salaries was stated to be an important retention factor. The existence of the private health facilities that offer part-time jobs varied from many in District A, a few in District B to none in District C.

*‘I work here and at two other private places [he mentions them] after my normal working hours…. I earn more than twice my salary from the private practice…. Where else should I go?’ [medical doctor-District A]*

In all three districts, the managers and the MDs interviewed felt that their work was not appreciated or supported by political leaders and the general community. In District A, political leaders were reported to be at the frontline in complaining about poor health services, regardless of the fact that they were not supportive when funds for improving the health services were requested during the meetings that they attended. In District C, the community used their community leaders to embarrass the MDs for not being able to provide medicines or supplies while they know the status of funding that was allocated for the health sector through the meetings they attend. All these together were reported to discourage the MDs and hence they felt the need to relocate to other places.

‘*You tell the relatives that we currently do not have the medicine [he mentions some] needed for the treatment of your patient. We advise them to go and buy it…. While you are sitting waiting for the medicine, they come back with the political leader…. You are embarrassed, but in the end, the leaders are the ones who set low budgets for you…. I am only here because an opportunity to leave has not yet arisen.*’ [medical doctor-District C]

The presence of better schools for their children, quality health care, and reliable transport systems was mentioned by participants from District B and C to be important contributing factors for them to stay. District A being in the largest city in the country was advantaged in having many good private schools and facilities for quality health care and education that are absent in district B and C.

*‘Education is not a priority here… There are no good private schools where you can take your children… The good hospital is this one, which, as I told you, lacks so many things … I will have to leave for another place where my children will have the assurance of good education, health care, and the likes…’ [medical doctor-District B]*

Furthermore, participants from District C added that transport to Dar es Salaam and elsewhere was a serious challenge as the only reliable means of transport was by flight, which was not affordable to most of them.

*‘My friend, we are very close to Dar, only 25 minutes, but yet we are very far from it. You need to be very rich to go there regularly… This adds to my reasons for not staying here anymore…’ [medical doctor-District C]*

### Strategies used by managers to retain doctors in situations of resource scarcity

Knowing the challenge of retaining the MDs in their districts, managers devised various strategies to ensure the retention of MDs. These included: establishing career development plans to address the challenge of career growth for MDs; the establishment of a minimum financial incentive package; and opening avenues for private practices in the district hospitals. The managers were, however, constrained by the scarcity of financial resources for equally competing priorities.

To ensure career development for the MDs, two of the three districts involved, B and C, had prepared written career development plans. The existence of a career development plan helped these districts to budget for their implementation annually.

‘*You know, MDs are a very fluid cadre…. If you do not show them their career path, be sure they will leave immediately; knowing this, we have the career development plan in the incentive package plan…. This has helped us to retain most of our MDs…*’ [FGD member-District A]

The establishment of a minimum financial incentive package was another strategy revealed by the managers in their effort to retain the MDs in the three districts. The common form of allowance that was found in all minimum financial packages of the three districts was the on-call allowance. In District B, on top of the on-call allowance there was the housing allowance, and in District A the minimum financial incentive package was comprised of an on-call allowance, a housing allowance, a uniform allowance and a responsibility allowance for head of sections (Table 2).

Managers from all three councils reported that being aware of the accommodation challenge; they made some form of arrangements in an attempt to overcome it. In Districts A and B short-term plans involved paying the subsistence allowance and the housing allowance early. In District C arrangements were in place to accommodate the incoming MDs in the homes of the available health workers before they secured houses for rent. We found that only District A had a long-term accommodation plan. This involved the building of hostels for the intern doctors that will not only cater for the interns’ accommodation but will also act as a source of funds for financial incentives..

‘*You know we have so many plans… We are planning to build a hostel for the intern doctors at our plot in [mentions the name of a neighbourhood place]. We will put everything there …they will pay less rent compared to if one is renting as an individual in the community… We will also provide reliable transport to travel to and from the hospital … We are sure this will solve the accommodation issue, motivate our interns and also generate income for the hospital…*’ [FGD member-District A]

In order to create opportunities for the MDs to earn extra income but still be retained at the district hospitals, we found that District A was in an arrangement to open an intra-mural private practice within all its hospitals; there were no such similar plans in Districts B and C.

‘*We are planning to create a window for a fast-track clinic (private practice) at our hospitals in [mention of two hospitals]… This will generate income for our hospitals, but at the same time raise incentives for our health workers and hence retain them…*’ [health manager-District A]

We found that regardless of the efforts made by the managers to retain doctors, the implementation was challenged by limited financial resources in areas of competing priorities. The informants reported that the districts had many other competing issues, such as the purchase of drugs and supplies, sustained supervision of facilities controlled by the districts, and provision of incentives not only to MDs but also to all their workers.

‘*We would like to pay all allowances on time…but we lack funds… If it were you, and you do not have drugs at the hospital and you get some amount of funds, what will you place high up in your priorities? Will you pay for allowances or purchase drugs?*’ [health manager-District B]

## Discussion

We aimed to analyse the experiences regarding the retention of MDs in the districts based on the perspective of MDs and the district health managers. Our study has found that the retention of MDs in the districts is contingent to working conditions, supportive living conditions in the community and the day-to-day strategies used by health managers to retain the MDs in resource-constrained situations.

The promise of a postgraduate scholarship after three years has been among the incentives offered to doctors who go to rural areas in Tanzania. However, in most cases the implementation of this incentive has failed [16, 33]. This is different from many parts of the world, where doctors are bonded to practise in the public sector and rural areas for a defined period as a condition of a postgraduate fellowship award and the incentive is fulfilled [34]. In Tanzania the concept of bonding the MDs is not new [21]. The MDs in Tanzania are bonded to practise within the country for a minimum of five years, however, they are not bonded to work in any specific setting or sector [21].

According to Shemdoe et al. in 2016, MDs are the hardest group to retain at the district level in Tanzania [35]. The government of Tanzania states that a weak management capacity across all levels in the country is one of the major factors of the poor retention of HRH [16]. Health managers are the key decision-makers and central planners of all health-care programmes at the district level [22, 23]. Most of the activities carried out by health managers on a daily basis in the handling of issues related to staff motivation (for example, supervision, payment of allowances, recognition of work performance) contribute to the retention or migration of MDs to other places [36].

### Establishment of a uniform financial incentive system across districts

As revealed by our study, similarly, financial incentives are widely recognized for their role in retaining physicians in rural areas in many other parts of the world [5, 37–40]. Despite the recognition of the role of financial incentives in the retention of doctors in rural areas, the existence of non-uniform financial incentives, as revealed by our study, may fuel the internal brain drain of MDs from one district to another where there are good financial incentives, among other factors. This finding is also in line with what Dambisya [41] stated in 2007, that the majority of East, Central and Southern Africa countries had forms of financial incentives, but their implementation has been a major impediment in retaining HRH in rural areas.

### Improving working and living environments

The unfriendly working conditions characterized by underequipped health facilities, shortage of supplies, and poor infrastructure resulted in dissatisfaction among the available MDs, thus fuelling their intention to leave. As revealed by our study, deserved attention is needed to properly equip the available health facilities to match the level of training of the MDs and thus add to their job satisfaction.

Our study also revealed that the difficulty in securing houses for rent and feelings of not being valued by the community made MDs feel unsettled, and hence their desire to relocate. This is similar to what Farooq et al. (2004) found in rural Pakistan, where the lack of good housing contributed to the emigration of doctors from rural areas [12]. Shemdoe et al. (2016) [35], documented that settling in the community was associated with the retention of the HRH in rural areas more than settling in their actual job posts. In rural Nigeria, social and physical support from the community contributed to the retention of HRH in rural and remote areas in the decentralized health sector [42]. WHO [38] considers personal support as an important policy recommendation in ensuring retention of HRH in rural areas. The 2008–2013 and 2014–2019 HRH Strategic Plans recognize the importance of a conducive working and living environment in ensuring low attrition of the available HRH in rural areas [16, 43]. However, the findings of this study hint that resources injected into the realization of this recognition are far too little to make this a reality. In Tanzania, experiences show the existence of some Non-Governmental Organizations that have managed to improve the working and living conditions by building houses for health workers in rural areas [44]; the government and other partners should team up in furthering these initiatives.

### Ensuring career growth of the MDs working in the districts

Lack of an assurance of career growth in the districts is among the pushing factors for MDs to leave the districts. Career growth has been found to be an important non-financial incentive in the retention of MDs in rural areas in many parts of the world [5, 39, 43]. In 2008, among the recommendations of a team of experts from Muhimbili University in a report submitted to Ministry of Health and Social Welfare was to ensure opportunities for career growth for HRH in rural areas [45]. Similarly, Shemdoe et al. (2016) documented that limited access to career opportunities was among the push factors for HRH to leave the rural areas in Tanzania [35].

### Addressing the question of social services in the districts

The absence of good schools for children, recreational facilities, an unreliable transport system and lack of good rental accommodation were found to contribute to MDs’ intention to relocate. Similar findings were documented by Farooq et al. (2004) [12], who wrote that among the reasons for the emigration of doctors in rural Pakistan were the poor social services in general, a lack of good schools for their children and a poor transport system. The role of recreational facilities in the retention of physicians in rural areas is also noted by Kotzee (2006) in rural Limpopo in South Africa [5]. However, addressing the question of social services is a multi-sectoral dimension issue. This requires addressing many questions beyond the health sector [38], such as support from other sectors like education, water, housing, agriculture, infrastructure, communication, and others. This view conforms to what the Global Health Initiative documented in 2004 – that overcoming the HRH crisis in poorly resourced countries is a shared responsibility and needs collaborative efforts [46].

To ensure the implementation of the shared responsibility, the Ministry of Health in collaboration with ministries of local government, education civil service management and finance, put in place a harmonized and implementable career growth plan for all districts.

The ministry responsible for local government in collaboration with ministries of civil service management and finance should harmonize the non-uniform financial incentive schemes across districts to ensure the existence of an effective and uniform financial incentive system across all districts. Stakeholders at the district level should come together to discuss the question of living conditions and social services in the districts, which is part of an important question of development requiring a multi-sectoral approach. Improvements in the availability of social services such as education, clean and safe water, and improved communication networks will subsequently attract individuals to invest in recreational facilities and real estate and finally contribute to the efforts to retain MDs who, like other human beings, are driven to areas where there is improved social well-being. Health managers, the community, and MDs should work together and discuss how to address the different context-specific c challenges arising that might fuel the attrition of MDs in their district.

#### Trustworthiness

Trustworthiness is attained in a qualitative study when the findings of such a study are worth believing [31]. Four criteria are used to assess the trustworthiness of a qualitative study; credibility, dependability, transferability, and conformability [47]. The credibility of the findings of this study was enhanced through the triangulation of informants with experiences and rich information on the study questions. In order to enhance the credibility and dependability of this study, triangulation of data collection techniques, study settings and researchers were used. Data were collected using interview guides, a focus group discussion guide, and document reviews were carried out in three different settings. In order to ensure that the findings reflected informants’ perspectives rather than the researchers’ understanding of the question under study, categories were inductively generated and presented with the support of subcategories and quotes. The transferability of the findings of this study is enhanced through the description of the study setting, context, data collection process, and analysis.

The fact that a medical doctor conducted the interviews and focused group discussions, might have introduced social desirability from the participants. However, the triangulation of data collection approaches and the fact that the interviewer was not senior but at the mid-level stage of his career offset this limitation. Finally, the findings of this study reflect the situation during the period in which data collection for this study was carried out.

## Conclusions

Assurance of career growth, provision of uniform minimum financial incentives and ensuring the availability of good social services and economic opportunities within the community across districts are among the important contributors for the retention of MDs across districts. The retention of MDs at the district level in a decentralized health-care system is a shared responsibility. It requires the contribution of many stakeholders both from the district level and from central government. We feel that it is high time for the government to devise a career path that may involve bonding doctors to practise at the district level for a defined period before awarding postgraduate education scholarships. However, the effectiveness of this strategy should be evaluated in light of the challenges faced in retaining doctors in the districts in Tanzania.

Although our study has pointed out the role of both financial and non-financial incentives in the retention of MDs in the districts in Tanzania, further studies are needed to establish the extent of these incentives that will appropriately ensure their retention.

## Additional file

Additional file 1: Interview guide for the interviews with District Health Managers (DHSS). This semi-structured interview guide (set of questions) was used for carrying the key informant interviews with the District Health Managers from the three districts. (DOC 34 kb)

## Abbreviations

CHMT: Council Health Management Team
DHSs: District Health Secretaries
DMOs: District Medical Officers
ECSA: Eastern, Central and Southern Africa
FGDs: Focus Group Discussions
HRH: Human Resources for Health
KIIs: Key Informant Interviews
MDs: Medical Doctors
SSA: Sub-Saharan Africa
WHO: World Health Organization
