# Supplementary material for: Retention of medical doctors at the district level: a qualitative study of experiences from Tanzania
Source: BMC Health Serv Res. 2018 Apr 10;18:260. doi: 10.1186/s12913-018-3059-0 (PMC5891935; doi:10.1186/s12913-018-3059-0)
Supplement: Supplementary file 1 — Interview guide for the interviews with District Health Managers (DHSS). This semi-structured interview guide (set of questions) was used for carrying the key informant interviews with the District Health Managers from the three districts. (DOC 34 kb) [file 12913_2018_3059_MOESM1_ESM.doc]

**APPENDIX III: INTERVIEW GUIDE WITH THE DISTRICT HEALTH MANAGERS**

**BEFORE STARTING THE INTERVIEW:**

- Salute all persons you meet in the interview venue (including those you don’t need for the interview)
- Introduce yourself to the interviewee
- Explain the purpose of your visit
- Request for consent to carry the study (provide him/her with a copy of the consent form and read it for him/her)
- Allow time for questions/discussion on the consent and clarification
- If satisfied request him/her to sign the consent certificate and return it to you (only the certificate)
- Switch on the digital recorder
- Thank him/her and then begin the interview

**Part I: Employment process**

1. Kindly tell us the employer of the medical doctors in your district (*Probe if all are under the government or from other health care sector partners*)
2. How do you host the newly posted/recruited medical doctors in part (I) above?
3. In your own words please tell us your experience on the reception of the medical doctors on the early days of their arrivals by other workers in your district (*Probe on cooperation, social support, materials, accommodation etc*)

**Part II: Retention of medical doctors**

1. In your own words, please tell us the available strategies to ensure that the recruited and posted medical doctors work and are retained in your district (*Probe on work, work environment, living environment, community support, local government support, financial and non-financial incentives etc*)
2. With the available strategies, please tell us the magnitude of migration of medical doctors from your district (*Probe on whether the magnitude has increased, decreased, where do they go [urban or rural, within the same region or different regions, within public or to private sector, if to private sector is it for the profit or not for profit, if not for profit is it in clinical works or public health?]*)
3. Based on your experience in this district, what causes migration of the medical doctors? (*Probe according to the responses*)
4. What is your comment on the available retention strategies? (*Probe whether they are enough, not enough and reasons for those feelings, and what are the plans to ensure retention in the future*)
5. From our talk and the available strategies and situation of medical doctors migration in your district and your experience of other districts in this country, what is your advice to the government and other partners regarding the retention medical doctors and HRH in general in your district and the country at large?
6. Thank you very much for the good responses to our questions, kindly we welcome you if you have any question/s or any concern that you would wish us to explain or address. (Give time for him/her to respond)
7. Finally, we have come to the end of our interview, once again thank you very much for your time and support. After our initial analysis, we may feel that we had forgotten or we may need more details on some of the responses and thus we may need to contact you again. Kindly bear with us and accept our visit or call to you again. In the event as well, you may remember something that you want to share with us or seek clarification from us, we are willing to talk to you anytime and most welcome.

**Part III: Identifications and socio-demographic profile of the informant:**

1. Name of the District ________________Ownership of district hospital______________
2. Job title___________________________ Position _____________________________
3. Age ________________________ Sex ___________________
4. Education level________________________ Field of study _____________________
5. Duration in current position __________________
6. What were you prior to this position? ________________________
7. Duration of working in this district __________________________
8. Working station prior to coming to this district _______________

**Thank you for your co-operation**
